# Clinical Characteristics, Management, and Outcomes of Intracranial Hydatid Cysts in Pakistan: A Narrative Synthesis

**DOI:** 10.7759/cureus.112472

**Published:** 2026-07-11

**Authors:** Faiqa I Khan, Omer B Adnan, Nusrat Fatima, Waleed Q Bashir, Ariba Qazi, Hamza Shabbir, Rimsha Khan, Muhammad Fawad Ul Hassan, Shoaib Ahmad, Sundas Irshad, Haseeb Mehmood Qadri

**Affiliations:** 1 Neurological Surgery, Punjab Institute of Neurosciences, Lahore, PAK; 2 Medicine, Mayo Hospital, Lahore, PAK; 3 Respiratory Medicine, Portsmouth Hospitals University NHS Trust, Portsmouth, GBR; 4 Medicine, Shalamar Medical and Dental College, Lahore, PAK; 5 Internal Medicine, Peshawar Medical College, Peshawar, PAK; 6 General Practice, Family Health Clinic, Jhelum, PAK; 7 General Practice, Akhtar Saeed Medical and Dental College, Lahore, PAK; 8 Neurological Surgery, National Hospital and Medical Center, Lahore, PAK; 9 Internal Medicine, Sheikh Zayed Medical College, Rahim Yar Khan, PAK; 10 General Surgery, Lahore General Hospital, Lahore, PAK

**Keywords:** albendazole, cysts, echinococcosis, headache, lung, meningitis, papilledema, parietal lobe

## Abstract

A hydatid cyst is caused by *Echinococcus granulosus*. It is transmitted to humans via the feco-oral route. It occurs mostly in the liver or lungs. Intracranial occurrence is a very infrequent phenomenon. This review aimed to provide an insight into the clinicopathological spectrum of primary intracranial hydatid cyst and its management in Pakistani patients. Databases (PubMed and Google Scholar) and national journals were searched using predefined keywords from their inception until October 31, 2025. All case reports (CR), case series (CS), and original articles that satisfied the inclusion and exclusion criteria were included. A total of 11 studies were included, reporting data on 86 patients.

The pooled mean age was 16.7 years, with a male predominance of 49 (59.03%). The median duration of illness was 12 (IQR: 6-12) months. The most frequently encountered symptoms were headache in 10/13 (76.90%), papilledema in 9/13 (69.20%), and motor deficit in 8/13 (61.50%). The most common location was supratentorial in 78/86 (90.70%), involving the fronto-parietal region in 21/83 (25.30%). Across the documented cases, the average diameter of the cyst was 8.81 cm. The lesion appeared hyperintense on T2 in six out of eight (75%) cases. Only 4/13 (30.79%) cases reported multiple cysts. Surgical management was opted for in all (86) cases. The Dowling technique was employed in 44 (51.16%), followed by excision in 41 (47.67%) cases. However, intraoperative cyst rupture was individually documented in three (23.07%) cases (from CR and CS). Postoperative meningitis was mentioned in only one CR. Medical therapy with albendazole was prescribed in nine (69.23%) cases, and it was continued for a mean of 28.80±28.89 weeks. The median duration of follow-up was 12 (IQR: 12-76) months. Recurrence was reported in three (23.07%) cases. Death was documented in 3/13 (23.07%) cases.

Meticulously performing the Dowling technique is safer to avoid intraoperative rupture. Subsequently, drastic complications can be prevented. Surgery, along with anti-helminthic administration, ensures the complete resolution of intracranial hydatid cysts. Long-term follow-up is necessary to investigate the course of the disease and achieve complete remission.

## Introduction and background

Cases of intracranial hydatid cyst occur worldwide, mostly in rural areas. Overall, it is endemic mainly in Northwest India, the Middle East, Australia, New Zealand, South America, and Central and Southern Europe [1]. Padayachy and Ozek reported that the majority of cases occur in the pediatric and young adult population [2]. Hydatid cysts occur primarily in the liver (75%) and lungs (15%) [3]. About 2% of all cases are intracranial, underscoring its rarity [2,4]. The liver and lungs can act as filters and often prevent the movement of the parasite to the brain [5]. Primary cysts arise from the direct lodging of larvae in the brain. These cysts are fertile and characterised by the presence of brood capsules. Secondary cases occur due to the rupture of a cyst elsewhere. In secondary cases, there is a greater chance of multiple cysts being present [3].

The clinicoradiological presentation of intracranial hydatid cyst mimics that of central nervous system (CNS) space-occupying lesions, and it can result in raised intracranial pressure which can be fatal. Moreover, the spillage of cyst contents is found to be associated with intracranial and spinal dissemination [5]. Din et al. reported that only 67% of the cases were preoperatively suspected to be hydatid cysts. Further, they added that merely nine patients showed up on follow-ups, among which four had recurrence and one died [5]. This emphasises the significance of probing into the differentials in order to ensure the accuracy of the management regimen, which is of utmost importance for clinicians, especially in agricultural lower-middle-income countries like Pakistan. 

Only a few cases from this geographical region have been reported over the decades. However, no comprehensive synthesis of data that represents the clinicopathological patterns and therapeutic relevance of the disease is available. This underscores the substantial gaps in the existing literature from this region regarding the disease's natural history. This review aimed to summarise the existing national literature and identify the knowledge gaps that could guide clinicians towards evidence-based practices, underscoring the importance of developing local guidelines.

## Review

Methodology

This narrative review was designed to provide a broad understanding of the management and outcomes of intracranial hydatid cysts in Pakistan. A comprehensive literature search was conducted by authors FIK and SI using electronic databases PubMed and Google Scholar from inception until October 31, 2025. The keywords used were "intracranial hydatid cyst Pakistan", "intracerebral hydatid cyst Pakistan", "Echinococcus granulosus", "intracranial hydatid disease", "Echinococcus", and "Pakistan" to identify relevant articles. The retrieved literature within the given timeframe was verified by author HMQ.

Inclusion Criteria

All cases reported in English as case reports (CR), case series (CS), and original research articles were included in this review. The study focused specifically on primary intracranial hydatid cyst cases documented in Pakistani patients. Individuals of all ages and both genders were considered eligible for inclusion. The selection of cases was further based on the neurosurgical management approaches employed in these patients, as summarised in Table 1.

**Table 1:** Search strategy for literature documenting intracranial hydatid cyst in the Pakistani population

Total publications identified using PubMed and Google Scholar	3976
Publications screened based on title relevance	1509
Publications excluded due to irrelevance	1491
Publications screened based on title and abstract	18
Full-text articles assessed for eligibility	15
Total studies included in the review	11

Exclusion Criteria

Non-English publications, letters to the editor, and conference abstracts lacking sufficient clinical details were excluded from the review. In addition, cases of hydatid cyst infections occurring outside Pakistan were not included, even if they were managed or treated in Pakistan. Cadaveric studies and animal-based experimental research were also excluded from the study.

Data Extraction

Data were extracted into a pre-formatted data sheet in Microsoft Excel 365 (Microsoft Corporation, Redmond, Washington, United States) and standardised for homogeneity. Information on demographic details, presenting features, examination findings, laboratory and radiological investigations, treatment, management protocol, follow-up, and outcomes was summarised.

Data Analysis

Continuous variables, such as age, number of patients, and treatment duration, were summarised using descriptive statistics. Categorical variables, including study type, residence, gender, history of animal contact, symptomatology, brain region involvement, imaging findings, management approach, intraoperative findings, evidence of dissemination, and patient follow-up and outcomes, were summarised using frequencies and percentages. Data was analysed using IBM SPSS Statistics for Windows, Version 24.0 (IBM Corp., Armonk, New York, United States) for descriptive statistical analysis. Due to the limited number of cases with substantially clinically relevant data, robust multivariate or pooled comparative analysis was not possible. 

Results

The total number of studies retrieved was 11. The articles consisted of six CR, two CS, and three retrospective cohorts. There was substantial heterogeneity in the available data. The total number of patients was 86. The demographic and management details of the 83 patients were available. There was a gender predilection towards males, 49 (59.03%), while females represented only 34 (40.96%). The pooled mean age (of the 83 patients) was 16.7 years. Individual age was documented in 13 cases. Most of the patients belonged to the pediatric age group, eight (61.53%). The studies included under review are summarised in Table 2.

**Table 2:** Reported cases of intracranial hydatid cyst from Pakistan pt.: patient; CR: case report; CS: case series; RC: retrospective cohort; SD: standard deviation; F: female; M: male; N/A: not available; PAIR: puncture, aspiration, injection, and re-aspiration *Predominant gender

Study	Study type	No. of pt.	Age/mean±SD (years)	Gender*	Management	Outcome
Din et al. [5]	RC	33	20	17F	Excision (medical N/A)	N/A
Razzaq and Hashim [6]	CR	1	20	M	Dowling + albendazole	Alive
Siraj et al. [7]	CR	1	11	F	PAIR + albendazole	Alive
Umerani et al. [8]	CR	1	22	F	Dowling + albendazole	Alive
Khan et al. [9]	CR	1	8	M	Dowling + albendazole	Alive
Ashraf et al. [10]	CR	1	10	M	Dowling + albendazole	Alive
Tariq et al. [11]	CR	1	16	M	Excision + albendazole	Alive
Mufti [12]	CS	4 (1st pt.)	14	F	Excision	Died
	CS	4 (2nd pt.)	9	M	Excision	Died
	CS	4 (3rd pt.)	22	M	Excision + mebendazole	Alive
	CS	4 (4th pt.)	10	M	Excision	Dead
Kibzai et al. [13]	CS	3 (1st pt.)	10	M	Dowling + albendazole + praziquantel	Alive
	CS	3 (2nd pt.)	40	M	Dowling + albendazole	Alive
	CS	3 (3rd pt.)	72	M	Dowling + albendazole	Alive
Azam et al. [14]	RC	37	12.4±1.2	23M	Dowling	N/A
Alam et al. [15]	RC	3 out of 50	N/A	N/A	Excision (medical N/A)	N/A

The data of only 13 patients, from CR and CS, were available. The history of domestic animal contact was mentioned in only one CR. The duration of illness was reported in 10 cases, which revealed a median of 12 (IQR: 6-12) months (mean 9.8±6.81). Four cases were reported from Balochistan, and one case was reported from each of the other provinces (Punjab, Sindh, and Khyber Pakhtunkhwa). The clinical details of the reported patients are given in Table 3.

**Table 3:** Clinical manifestations of the reported patients with intracranial hydatid cyst, where N is the total number of documented cases *Documented for 13 available cases

Variable (N=13)*	Percentage, % (n)
Headache	76.90 (10)
Papilledema	69.20 (9)
Motor deficit	61.50 (8)
Vomiting	53.80 (7)
Seizures	38.50 (5)
Fever	23.07 (3)
Eosinophilia	23.07 (3)

The size of the lesion was mentioned in only three CR and one cohort; the average diameter calculated was 8.81 cm. The multiplicity of cysts was reported in only four (30.76%) cases. Among 13 cases, the lesion was bilateral in only one (7.69%). The lesion's predilection towards the right side was noted in nine (69.20%) and left in only three (23.10%). Magnetic resonance imaging (MRI) was used as the primary investigation in eight cases. Among these, the cyst lesion appeared hypointense on T1 in six (75%) and hyperintense on T2 in six (75%) cases. The perilesional oedema on MRI was documented in two (25%). A non-enhanced cyst on contrast MRI was observed in six (75%). Computed tomography (CT) without contrast was employed in three cases. The lesion appeared hypodense in three cases; however, calcification was reported in none (Table 4).

**Table 4:** Radiological location of the cyst(s) in the reported patients with intracranial hydatid cyst, where N is the total number of documented cases *The exact location of the lesion was missing in three cases

Variable	Percentage, % (n)
Anatomical region of the brain (N=86)	
Supratentorial	90.70 (78)
Infratentorial	9.30 (8)
Functional region of the brain (N=83)*	
Fronto-parietal	25.30 (21)
Parietal	21.70 (18)
Parieto-occipital	18.10 (15)
Frontal	10.80 (9)
Cerebellum	9.60 (8)
Temporal	3.61 (3)
Temporo-parietal	3.61 (3)
Fronto-temporo-parietal	2.41 (2)
Occipital	2.41 (2)
Temporo-parieto-occipital	1.20 (1)
Fronto-temporal	1.20 (1)

Surgical intervention was employed in all 86 patients (Table 5). However, the individual management regimen was documented in only 13 cases. The spillage of cyst content was mentioned in three (23.07%) cases. All intraoperative ruptures were managed with copious saline irrigation. Postoperative meningitis was only mentioned in one (7.69%) CR. Recurrence was only reported in three (23.07%) cases.

**Table 5:** Management and outcomes of the reported patients with intracranial hydatid cyst, where N is the total number of documented cases *N is different for all variables

Variable	Percentage, % (n)
Surgical management (N=86)*	
Dowling technique	51.16 (44)
Excision	47.67 (41)
Puncture, aspiration, injection, and re-aspiration	1.16 (1)
Medical management (N=13)*	
Albendazole	69.23 (9)
Mebendazole	7.69 (1)
Praziquantel	7.69 (1)
Mean duration of therapy in weeks (N=5)*	28.80±28.89
Mean duration of follow-up in months (N=11)*	12.05±14.83
Outcome (N=13)*	
Alive	76.92 (10)
Expired	23.07 (3)

Discussion

Intracranial hydatid cysts are caused by cestodes (tapeworms) of the genus *Echinococcus*. Genotypes G1 and G3 (commonly associated with sheep) are the most common. Genotypes G8 and G10 have a northern Holarctic distribution, while genotypes G6 and G7 have been widely reported across Eurasia, the Middle East, and Africa [4]. *Echinococcus granulosus* is the most commonly occurring infectious organism in its larval form [5]. The organisms reside in the small intestine of their definitive host (typically dogs and other canids) before releasing eggs in the feces. Once ingested by intermediate hosts (humans, sheep, camels, etc.), the eggs release oncospheres in the small intestine of their host. These oncospheres travel throughout the body and can lodge in different organs, forming hydatid cysts [5]. Usually, the liver and lungs filter them, and intracranial cases only occur when the larvae make it past both organs.

In our study, 8/13 (61.53%) of the patients were under 18 years, meaning that the majority of patients belonged to the pediatric population. This was consistent with previously recorded literature [16,17]. The pooled mean age of our patients was 16.7 years. Out of the 83 patients included in this review, 49 (59.03%) were males, underscoring its prevalence in men. This may be related to the rural predisposition of the disease and greater exposure to fauna as a result of activities like rearing and hunting. The transmission cycle primarily involves animals, and humans serve as the terminal host [4,16].

From the reported sector of Pakistan, 4/13 (30.76%) patients were from Balochistan, and one patient was from each of the other provinces. Although Punjab is the most agriculturally productive province, the reported data lack any significant representation from this region. The authors speculate this could be attributed to the developed veterinary practices of Punjab as compared with those of the other provinces. Moreover, animal and host encounters are more common in rural areas, rather than in agricultural regions. However, in our review, only one CR mentioned a domestic animal encounter. An analysis of literature verifies the predominance of this disease in agricultural and livestock-rearing regions of the world [2-5].

Our findings indicated the multiplicity of cysts in 4/13 (30.76%) patients. Most occur in the supratentorial location, 78 (90.70%).This is supported by the cerebrum's large blood supply, especially the middle cerebral artery (MCA) and its terminal branches [4,18]. Among the specific regions, we found the cyst was more common in the fronto-parietal, 21 (25.30%), followed by the parietal, 18 (21.70%), and parieto-occipital, 15 (18.10%), lobes. This predilection was supported by global texts, which show predominance in the parieto-occipital lobes (10.92%) [2,4,5]. In our review, cysts were mostly found to be on the right cerebrum in 9/13 (69.20%) cases. This could be more of an observational than a causal relationship between right-sided dominance and hydatid cyst laterality. As described by Sharma et al. in a meta-analysis, there is no calibre difference in right or left MCAs [19]. A slight reference can be drawn with the slightly significantly larger right hemisphere [20]. However, in a recent systematic review, involvement of the left multilobe (28.1%) was more than the right multilobe [21]. Rarely, a cyst is found in other sites of the CNS, such as the pons, brainstem, periventricular area, middle fossa, cerebellopontine angle, thalamus, parasellar regions, interpeduncular cisterns, cavernous sinus, cerebellum, and ventricles [5,22]. Brain hydatid cyst (BHD) can be primary or secondary [21]. Primary are rare, as they occur in the brain without prior other organ involvement; they are usually unilocular. Secondary are multiple cysts, and they occur due to the rupture of the primary cyst in other organs of the body [21]. We found four (44.40%) multilobular, but none were secondary. Another reason for multiple intracranial cysts could be the ingestion of multiple eggs or cyst dislodging from other organs, especially in the case of left-to-right shunting (patent ductus arteriosus, etc.) [17].

The most frequent presenting symptoms were headache in 10/13 (76.90%), papilledema in 9/13 (69.20%), motor deficit in 8/13 (61.50%), vomiting in 7/13 (53.80%), seizures in 5/13 (38.50%), and fever in 3/13 (23.07%) of the patients. The clinical presentation was consistent with international literature [4]. These signs of increased intracranial pressure stem from the increasing mass effect as the lesion slowly grows in size (1.5-1 cm per year), reaching 4-5 cm or even 7 cm as noted internationally [22,23]. The average diameter in our studies was 8.81 cm, which is almost consistent with the literature. However, some cysts, called giant cysts, may present at even larger sizes as reported in previous Pakistani literature. Examples include a 22.5 cm and a 15 cm, diagnosed in Karachi and Abbottabad, respectively [5,12]. In spite of being a parasitic infection, fever and eosinophilia were reported in as few as 3/13 (23.1%) of our patients. Hydatid cysts are assumed to contain sterilised fluid, surrounded by a thick capsule that prevents antigens from entering the host immune system and eliciting an immune reaction [24].

Radiological diagnosis of cysts of *Echinococcus* is adequately done by ultrasound imaging, MRI, or CT scan. For cysts in the liver, lungs, and sites other than the brain, ultrasound imaging is preferred due to its availability, high resolution for diagnosis, and lack of radiation exposure. Moreover, ultrasound also plays a role in the classification of these cysts according to the official criteria of the World Health Organization (WHO), namely, (1) active group (CE1: unilocular; CE2: multivesicular), (2) transitional group (CE3a: detachment of endocyst membrane; CE3b: predominantly solid cysts), and (3) inactive group (CE4: having solid content; CE5: with calcification) [25]. However, in cases of intracranial cysts, CT scans and MRIs are preferred as ultrasound imaging has proven to be unsatisfactory [22]. On CT scans, these cysts are observed as hypodense, rounded mass lesions without perilesional oedema. MRIs show them to be smooth, well-defined, thin-walled, homogenous spherical lesions [2]. Moreover, T1WI images often reveal hypointense lesions without any contrast enhancement and oedema, whereas T2WI images most often demonstrate hyperintense lesions, findings which are consistent with our results, due to the high water content of the cyst [2,18]. Perilesional oedema is rare and was reported in 2/8 (25%) of our patients; however, calcification was reported in none, as supported by the literature [2]. Calcification is thought to be found in cases of cyst rupture, as indicated by irregular walls, or in cases of parasite death [2]. Oedema also happens when cyst content elicits the host immune system, which, again, is barely seen, as cysts have a very protective wall surrounding their fluid content [2,24].

Surgical excision has proven to be the most effective method of cyst removal with the best prognosis. The Dowling technique is most widely used internationally, which was employed in 44 (51.16%) of our patients. It involves forcing normal saline around the cyst, following craniotomy, and basically pushing it out using hydrostatic expulsion. Multiple cysts, deep-seated location, and adhesion to surrounding structures make it challenging, and alternative options are explored [21]. This requires advanced surgical techniques like microsurgery or navigation guidance surgery. Another is the puncture, aspiration, injection, and re-aspiration (PAIR) method, used in only 1/13 (1.16%) of our patients. It is reserved for problematic lesions for which the Dowling technique is insufficient, such as cysts in deep regions or eloquent high-risk areas [7,23]. An older method, simple surgical excision, was also done in 41 (47.67%) of our patients. The literature also supports burr hole and needle aspiration of the cyst followed by capsule removal [21]. Moreover, variations in the precise site of the lesion, such as in the cortical, subcortical, or ventricular regions, can also cause variations in the surgical approach [18]. The most common challenge during surgery is cyst rupture, leading to the spillage of the cystic fluid. This can lead to recurrence (reported in 30% of our patients), fatal anaphylactic shock, and meningitis [7,23]. Two methods have been documented to manage this complication: hypertonic saline irrigation and medical therapy via albendazole. The first one needs vigilance and care so as to avoid systemic absorption of the hypertonic saline. Our results demonstrate that all our cases of rupture were managed by hypertonic saline irrigation; a review of existing Pakistani and global literature proves that both methods are used in clinical practice [5,18]. Other risks include hematoma, hygroma, or oedema filling the dead space left after the excision; however, we did not encounter any of these in our review. Only two had postoperative meningitis. Recurrence is also a concern, especially in cases of primary cyst rupture, as the primary cyst is fertile [21].

Medical therapy for cysts of *Echinococcus *includes anti-helminthic drugs such as albendazole (most common), mebendazole, and praziquantel. These are used for inoperable cases, intraoperative cyst rupture, the prevention of recurrence, and hydatid dissemination [2]. Albendazole sterilises the cyst, hence decreasing the immune reaction [26]. In our review, 11/13 patients received additional medical treatment. Albendazole was given in 9/13 (69.23%) and mebendazole in 1/13 (7.69%). However, praziquantel was prescribed in 1/13 (7.69%) cases. The median duration of therapy was 12 (IQR: 8-56) weeks, which approximates to almost six months; we were unable to extract any long-term complication rate in our population. The amounts given in Pakistan and globally, according to the recommendations of WHO, are 10-15 mg/kg/day in divided doses for 3 months continuously or for recurrent 1-month courses, with an interval of 15 days after each month [2,5]. Combined surgical and medical therapy has so far proved to be the cornerstone of significantly improved prognostic outcomes [17, 25]. Generally, the success rate for cyst removal via surgery is very good, especially if they were removed unruptured. The median duration of follow-up in our patients was 12 (IQR: 12-76) months, with 10/13 (76.90%) of the patients alive at the last follow-up. This positive outcome is also advocated by global literature [5,17].

Limitations

Intracranial hydatid cyst is already a rare occurrence, making the count of eligible studies very limited. We would like to point out that the documentation of cases of intracranial hydatid cysts in Pakistani biomedical journals was poor, with large heterogeneity in data; hence, a Preferred Reporting Items for Systematic Reviews and Meta-Analyses (PRISMA)-based systematic review for a handful of cases was not feasible. We opted for a narrative review with descriptive analysis to at least study the features and trends of this disease in the Pakistani population, which is a baseline step to conduct more organised studies globally in the future. There was substantial heterogeneity in documented variables across the included studies. Most articles were CR, and the proper methodological reporting (including history of animal contact, intraoperative complications, presence of any immunosuppressing condition, post-rupture screening, early and late follow-up, etc.) was lacking, making it difficult to synthesise data for any inferential statistics. Many case reports lacked long-term follow-up data. This prevented the assessment of whether surgical excision followed by medical therapy was sufficient for the complete eradication of the disease. To synthesise substantial evidence-based data, more studies with proper documentation of medically vs. surgically treated cases or intact vs. ruptured cyst outcomes are needed. Furthermore, documenting the region was limited, which does not enable us to draw any conclusive evidence of the endemic area in Pakistan.

Recommendations

There is a scarcity of published literature as well as difficulty in procuring and saving follow-up data, both in Pakistan and in other endemic regions of the world. It is therefore important to stress the need for multicenter longitudinal studies to generate a more comprehensive database. Lowering the rates of this helminthic infection in endemic countries like Pakistan can be achieved by developments in the timing of diagnosis, neurosurgical management, multidisciplinary collaboration, and providing a standardised management protocol. Such advances will improve the quality of care and will also aid in collecting data in a systemised way, which, when coupled with multicenter data collection and pooling with other countries, will unlock broader horizons for collaborative research and advancement of management techniques. Moreover, in diseases like these, veterinary healthcare should be of concern to prevent vector transmission. Microsurgical and navigation-guided techniques should be employed where necessary to prevent vital structure damage and delivery of the cyst without rupture. Adherence to anti-helminthics must be ensured to prevent recurrence and achieve complete eradication.

## Conclusions

Intracranial hydatid disease remains a rare but notable public health concern in developing countries like Pakistan, especially in their agricultural zones and socioeconomically under-resourced areas. The disease frequently involves the supratentorial region, with a clear unexplained predilection for the frontal and parietal lobes. The clinical presentation of intracranial hydatid cysts varies widely owing to the functional area of the brain it seeds in. Growing cysts become large enough to cause mass effects. Meticulous execution of the Dowling technique to avoid intraoperative cyst rupture, excision of the cyst with capsule, and concomitant anti-helminthic therapy may facilitate the complete resolution of intracranial hydatid cysts.
